# Key Factor Regulating Inflammatory Microenvironment, Metastasis, and Resistance in Breast Cancer: Interleukin-1 Signaling

**DOI:** 10.1155/2021/7785890

**Published:** 2021-09-23

**Authors:** Fengjie Liu, Lihong Li, Meng Lan, Tengteng Zou, Zhaodi Kong, Tiange Cai, Xiao Yu Wu, Yu Cai

**Affiliations:** ^1^College of Pharmacy, Jinan University, Guangzhou 510632, China; ^2^College of Life Sciences, Liaoning University, Shenyang 110036, China; ^3^Leslie Dan Faculty of Pharmacy, University of Toronto, Toronto, Canada M5S 3M2; ^4^Guangdong Key Lab of Traditional Chinese Medicine Information Technology, Guangzhou 510632, China

## Abstract

Breast cancer is one of the top-ranked cancers for incidence and mortality worldwide. The biggest challenges in breast cancer treatment are metastasis and drug resistance, for which work on molecular evaluation, mechanism studies, and screening of therapeutic targets is ongoing. Factors that lead to inflammatory infiltration and immune system suppression in the tumor microenvironment are potential therapeutic targets. Interleukin-1 is known as a proinflammatory and immunostimulatory cytokine, which plays important roles in inflammatory diseases. Recent studies have shown that interleukin-1 cytokines drive the formation and maintenance of an inflammatory/immunosuppressive microenvironment through complex intercellular signal crosstalk and tight intracellular signal transduction, which were found to be potentially involved in the mechanism of metastasis and drug resistance of breast cancer. Some preclinical and clinical treatments or interventions to block the interleukin-1/interleukin-1 receptor system and its up- and downstream signaling cascades have also been proven effective. This study provides an overview of IL-1-mediated signal communication in breast cancer and discusses the potential of IL-1 as a therapeutic target especially for metastatic breast cancer and combination therapy and current problems, aiming at enlightening new ideas in the study of inflammatory cytokines and immune networks in the tumor microenvironment.

## 1. Introduction

The history of interleukin-1 (IL-1) dates back to the early 1940s, from the identification of the fever-inducing activity of “soluble factors” produced by endotoxin-stimulated leukocytes, to the discovery of inflammasomes and clinical benefits of anti-IL-1*β* therapy, encompassing the entire field of inflammatory cytokines, Toll-like receptors (TLRs), and innate immune responses [1]. IL-1 includes two agonists, IL-1*α* and IL-1*β*, which trigger signals via binding to IL-1 receptor 1 (IL-1R1) and recruitment of an accessory peptide chain [2]. The reason for having two IL-1 agonists may lie in the difference in robustness or specific functions between them [3]. Subsequently, the IL-1 receptor antagonist (IL-1Ra) was discovered, which specifically blocks IL-1R1 [4]. IL-1, together with several other sequentially discovered structurally related members, constitutes the IL-1 family. To date, there are 11 members of the IL-1 family [5]. These cytokines have pleiotropic functions, including regulating innate and adaptive immune responses; participating in the physiological regulation of homeostatic processes and host defense against pathogens, injury, and environmental stresses; and directly affecting transcription of mRNA [6, 7]. Antagonists in the IL-1 family cytokines and inhibitors in the IL-1 receptor family that function as membrane-bound or soluble decoy receptors have an important role in the biological activity of IL-1 and the negative regulation of inflammation induced by IL-1 [8].

IL-1 is a proinflammatory cytokine that affects cellular and organ inflammatory reactions, immune responses, and homeostatic regulation at low concentrations, and has long been known to be important in oncogenesis, invasion, metastasis, and tumor host interactions [9, 10]. IL-1 blockers applied to some autoimmune and inflammatory diseases are currently being tested in preclinical and human clinical experiments for tumor therapy [11]. The role and regulatory mechanisms of IL-1 signaling has been extensively studied in a variety of infectious diseases, inflammation, and inflammation-related cancers, such as colon cancer, liver cancer, gastric cancer, cervical carcinoma, and lymphoma, but is still not well understood in breast cancer (BC) [12–14]. Although BC has not been recognized as an inflammation-related cancer except inflammatory breast cancer (IBC) [15], it is well known that inflammation is a fundamental feature of the tumor microenvironment (TME) [16]. TME infiltrated a large number of immune and inflammatory mediators, including abundant IL-1 cytokines derived from immune or tumor cells. These mediators were thought to be key regulators of TME [17, 18]. Studies in recent years have also confirmed the important role of IL-1 in the BC microenvironment. However, the role of IL-1 signaling in the BC microenvironment is controversial, despite most studies showing its tumor promoting effects [19]. The dual functions in tumorigenesis, both pro- and antitumorigenic, largely depend on the source of the cytokines, levels present in TME, tissues and organs involved, inflammatory context, and stage of the cancer [20].

No matter which subtype of BC, it shows different degrees of inflammatory status in cancer progression, which is a common denominator, and thus can provide a generally applicable therapeutic idea. Whether IL-1 is the originator of the protumor inflammatory microenvironment in BC remains unclear. Questions on the regulatory mechanisms of IL-1 signaling; the crosstalk network between different cells and between different intracellular signal transductions, by which IL-1 signaling and its regulation affect aspects of inflammation, immunity, metastasis, and drug resistance in BC microenvironment; and the usability of IL-1 signaling blockade in terms of clinical treatment in BC may lead us to discover a novel biomarker or effective therapeutic target.

## 2. Breast Cancer and Targeted Therapy

In the last decade, the global incidence of BC has shown an increasing trend with no significant reduction in mortality [21, 22]. Patients with early, locally advanced, and locally recurrent BC are considered to have a higher chance of cure. But nearly 12% of patients diagnosed with BC will eventually develop into metastatic disease, which received palliative treatment only trying to relieve symptoms, prolong survival, and maintain quality of life [23–25].

Systematic treatment of patients with nonmetastatic BC is determined by subtypes: hormone receptor-positive (HR+) patients receive endocrine therapy, and a few of them receive chemotherapy at the same time; human epidermal growth factor receptor 2-positive (HER2+) patients receive HER2-targeted antibody or small molecule inhibitor combined chemotherapy; due to the high heterogeneity, invasiveness, and lack of treatment options, chemotherapy is still the standard treatment for triple negative breast cancer (TNBC). Local treatment of nonmetastatic BC includes surgical resection and postoperative radiotherapy [26, 27]. In recent years, the progress of chemotherapy, endocrine therapy, immunotherapy, new targeted therapy, and combination therapy has significantly improved the clinical outcomes and prognosis of BC, and made the prospect of long-term disease control of metastatic BC more and more realistic [28–31]. However, acquired tumor resistance is the major reason limiting the treatment effect [32]. Therefore, great efforts have been devoted in recent years to evaluate the molecular characteristics of metastasis and elucidate the mechanisms of drug resistance in BC in order to find novel molecular targets and therapeutic strategies [33–35].

Targeted therapies locate and inhibit tumor-related pathways, such as phosphoinositide 3-kinase (PI3K)/V-akt murine thymoma viral oncogene homolog (AKT)/mammalian/mechanistic target of rapamycin (mTOR), rapidly accelerated fibrosarcoma (RAF), mitogen-activated protein kinase (MAPK), histone deacetylase (HDAC), cyclin-dependent kinases (CDK), and poly(ADP-ribose) polymerase (PARP), by molecules binding to extracellular receptors, such as trastuzumab against HER2 and bevacizumab against vascular endothelial growth factor (VEGF), or by cytoplasmic blocking of small molecules, which is mainly aimed at the tumor [36–38]. Targeted therapies hold good promise in cancer treatment. But targeted therapies for BC face the challenges of diminishing returns, increasing costs of cancer care, and risk of overtreatment [39]. TME is associated with proliferation, angiogenesis, metastasis, apoptosis inhibition, immune system suppression, and drug resistance in BC [40]. Due to the recognition that the cancer stroma is the protagonist of cancer progression and the fact that TME is much more genetically stable than cancer, the development of dual anticancer strategies that target both cancer cells and TME will undoubtedly become the focus of current and future research, which may also be the key to the treatment success of BC with genetic and phenotypic heterogeneity [41].

## 3. Breast Cancer Microenvironment

In addition to tumor cells, the BC microenvironment also contains a large number of other distinct cell types collectively referred to as stromal cells, including vascular endothelial cells (VECs), cancer-associated fibroblasts (CAFs), mesenchymal stem cells (MSCs), and immune cells such as tumor-associated macrophages (TAMs), myeloid-derived suppressor cells (MDSCs), T lymphocytes, B lymphocytes, as well as myoepithelial cells and adipocytes. Besides, several noncellular components, including extracellular matrix (ECM), exosomes, soluble cytokines or signaling molecules, and occasionally the blood and lymphatic vascular network, have been identified. The physical characteristics of TME, such as hypoxia, acidity, inflammation, and high interstitial fluid pressure, also differ from those of normal tissues [42]. TME is now recognized as a hallmark of cancer biology. Infiltration of TAMs, tumor-associated neutrophils (TANs), MDSCs, T regulatory cells (Tregs), T helper IL-17-producing cells (Th17s), metastasis-associated macrophages (MAMs), and CAFs enables immune escape, tumor growth, angiogenesis, metastasis, and treatment resistance in multiple tumors, including BC [43–45]. Moreover, the ECM, ECM proteins, chemokines, cytokines, growth factors, and the physical state of TME can all influence the behavior and treatment response of solid tumor in complex ways and predict clinical outcomes [35, 46, 47].

The relevance of inflammation to cancer has been demonstrated. Normally, proinflammatory and anti-inflammatory signals are maintained in a state of balance called inflammatory homeostasis (central). However, dysregulation and prolonged maintenance of inflammation lead to chronic inflammation or immunosuppression and may contribute to the development of several diseases, including cancer [48]. Tumor itself and tumor-triggered inflammation can also promote malignant progression and cause immunosuppression through the recruitment and activation of inflammatory cells [49]. Complement system and kinins, vasoactive amines, cytokines, and hormones are considered important inflammatory mediators in the BC microenvironment [50]. Various overexpressed inflammatory mediators exert their biological effects locally or at a distance through the systemic circulation to influence progression, metastasis, and treatment outcome of BC by establishing a supportive immune or inflammatory microenvironment [51]. Compared with other cancers, the role of the immune and inflammatory systems in the development of human BC remains poorly understood. As the prototypical inflammatory cytokine upstream of the cytokine cascade, the role of IL-1 in tumor initiation and progression and tumor-associated inflammation is of sufficient interest [10]. The availability of IL-1R1 conditional demolded mouse models has made it possible to dissect the role of IL-1/IL-1R1 signaling in different cell types in TME [18].

## 4. IL-1 Single-Nucleotide Polymorphisms and Breast Cancer Risk

Genetic variation is an important inducer of cancer, and single-nucleotide polymorphisms (SNPs) are one of the most common heritable variations in the human genome. There has been an attempt to explore the association between IL-1 SNPs and cancer risk. 144 different SNPs have been described in the IL-1 *β* gene [52]. At base pairs in these transcription sites, base transitions between C and T have been widely reported [53, 54]. Recent studies have shown that IL-1*β* SNPs rs1143634, rs1143627, rs1143623, and rs10490571 were suggested to be associated with BC risk, while the results of the association of rs16944 with BC risk were inconsistent [55–62]. More studies on IL-1 SNPs and their functions affecting the balance of IL-1 protein may help to identify patients at risk and the severity of the disease and may provide additional therapeutic options in some groups of patients.

## 5. IL-1 Signaling

IL-1 signaling under the title of the article refers to intercellular crosstalk and intracellular signal transduction driven by the IL-1*α*/*β*-IL-1R system. IL-1 is derived from dendritic cells (DCs), monocytes, macrophages, mast cells, neutrophils, B cells, T cells, endothelial cells, epithelial cells, dying cells, and tumor cells [5]. As the initial member of the IL-1 family, IL-1 has been recognized as a key immune and inflammatory mediator with important roles in tumorigenesis, invasion, metastasis, and tumor host interactions by mediating chronic inflammation, tumor angiogenesis, activation of the IL-17 pathway, induction of MDSCs, recruitment of macrophages, and skewing and suppression of antitumor immunity [9, 10].

The potent proinflammatory effects of IL-1 follow three major steps: cellular expression, membrane receptor binding, and intracellular signal transduction. IL-1*α* and IL-1*β* are translated into 31 kDa precursor forms (pro-IL-1*α* and pro-IL-1*β*), cleavage of which generates 17 kDa mature forms (IL-1*α* and IL-1*β*). Unlike pro-IL-1*β*, pro-IL-1*α* has a functional nuclear localization signal in the N-terminal domain [63, 64]. Thus, both forms of IL-1*α* are biologically active and have dual functions, i.e., binding to IL-1R1 to exert damage-associated molecular patterns (DAMPs) or “alarming” function, or directly regulating transcription of genes [20, 65]. It was found that HS-1-associated protein X (HAX) 1, a protein associated with mitochondria, endoplasmic reticulum, and nuclear membrane, can bind to pro-IL-1*α* and promote its nuclear localization. Pro-IL-1*α* interacts with histone acetyltransferases P300, p300/CBP-associated factor (PCAF), and general control nonrepressible 5 (GCN5) in the nucleus and regulates gene expression independently of IL-1R. Pro-IL-1*α* is also posttranslationally modified, including myristoylation at Lys82, phosphorylation at Ser90, and glycosylation at D64. Myristoylation and glycosylation are associated with the membrane-bound form of IL-1*α*. But the functions of these modifications are largely unknown [63]. The production of IL-1*α* requires intracellular or extracellular proteases (calpain II, caspase-1, chymotrypsin, elastase, and granzyme B) [63, 64]. The necessity of this proteolytic cleavage may manifest in the enhanced biological potency of pro-IL-1*α* cleaved by inflammatory proteases [66]. IL-1*α* is constitutively expressed in epithelial, endothelial, and stromal cells and can be upregulated in hematopoietic and nonhematopoietic cells by a variety of stimuli, including Toll-like receptor (TLR) agonists, inflammatory cytokines, oxidative stress, fatty acid-induced mitochondrial uncoupling, and hormones [63]. IL-1*α* promoter lacks typical TATA and CAAT box regulatory regions but contains binding sites for activator protein-1 (AP1) and nuclear factor kappa B (NF-*κ*B) transcription factors, which are upregulated during inflammatory stimulation [63].

As a key proinflammatory cytokine, IL-1*β* is mainly expressed in innate immune cells [18]. Different from IL-1*α*, IL-1*β* is only active as a mature, secreted molecule, with tightly regulated processes of production and secretion. IL-1*β* requires a “dual signal” process to become activated. Signal 1 events represent the transcription and translation of pro-IL-*β* induced through activation of TLR, tumor necrosis factor (TNF), IL-1R, AP1, or NF-*κ*B. Signal 2 is an activation step dependent on the inflammasome complex, which consists of a sensing molecule NOD-like receptor (NLR)/AIM2-like receptor (ALR), an adaptor molecule apoptosis-associated speck-like protein (ASC), and an activation and recruitment domain of the caspase. The inflammasome platform recruits and activates caspase-1/11, which cleaves the N-terminal 116 amino acids of the pro-IL-1*β* polypeptide to convert it into mature IL-1*β* [63, 67–71]. NLR protein families mostly have a variable N-terminal domain and a C-terminal leucine-rich repeat (LRR) domain. This family is further divided into NLRP or NLRC receptors based on the presence of an N-terminal pyrin domain (PYD) or caspase activation and recruitment domain (CARD). Among them, NLRP1 (NOD-like receptor family PYD domain-containing protein 1), NLRP3, and NLRC4 (NOD-like receptor family CARD-containing protein 4) are able to induce the formation of an inflammasome, serving as platforms for activating caspase-1 [72]. However, inflammasome-independent processing of IL-1*β* has also been demonstrated in caspase-1/11-deficient mice, and neutrophil proteases including elastase, proteinase-3, granzyme A, and cathepsin G are able to extracellularly convert pro-IL-1*β* into active mature protein [65]. Since IL-1*α* and IL-1*β* lack a signal peptide, they are not secreted via the conventional endoplasmic reticulum/Golgi pathway but via an unconventional protein secretion pathway [73]. This mode of secretion may involve exocytosis of secretory lysosomes, cytolysis, multivesicular body formation, microvesicle shedding, and direct efflux during hypertonic cell death, and cleavage of IL-1*β* is thought to be necessary for this mode [65, 67].

**Figure 1:**
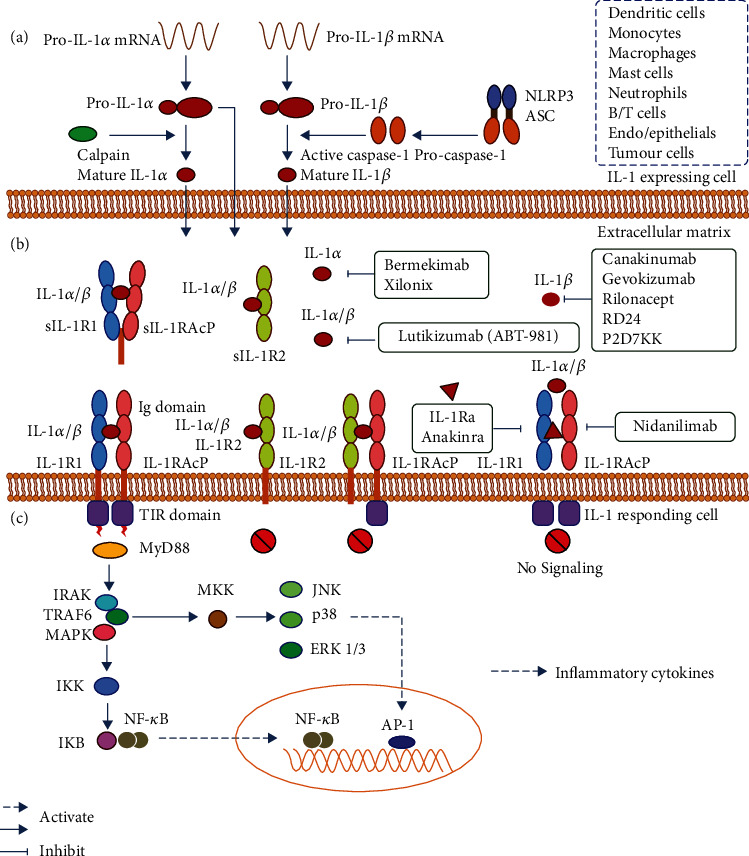
Typical IL-1 signaling. (a) Synthesis and secretion of IL-1*α* and IL-1*β*. (b) Several natural or recombinant biologics block IL-1 and its process of binding to membrane receptors. (c) IL-1 activated intracellular signaling. Abbreviations: IL—interleukin; IL-1R—IL-1 receptor; IL-1Ra—IL-1 receptor antagonist; IL-1RAcP—IL-1 receptor accessory protein; Ig—immunoglobulin; TIR—Toll- and IL-1R-like; NLRP3—NOD-like receptor family PYD domain-containing protein 3; ASC—apoptosis-associated speck-like protein; MyD88—myeloid differentiation primary response gene 88; IRAK—interleukin-1 receptor-associated kinase; TRAF6—TNF receptor-associated factor 6; MKK—mitogen-activated protein kinase kinase; JNK—c-Jun N-terminal kinase; ERK—extracellular signal-regulated kinase; MAPK—mitogen-activated protein kinase; IKB—inhibitor kappa B; IKK—inhibitor kappa B kinase; NF-*κ*B—nuclear factor kappa B; AP1—activator protein-1.

The IL-1 receptor family comprises 10 members, simply named IL-1R1~IL-1R10 [65]. The extracellular Ig domains of the receptors share the same structure with the intracellular Toll-like/IL-1R (TIR) domain [8]. IL-1*α* and IL-1*β* bind to the extracellular Ig domain of IL-1R1. Ligand-induced conformational changes recruit the nonbinding accessory chain IL-1RAcP to form a heterotrimeric complex [8]. The trimeric IL-1R complex recruits myeloid differentiation primary response gene 88 (MyD88) via its C-terminal TIR domains. MyD88 oligomerizes via its death domain (DD) and TIR domain, and it interacts with interleukin-1 receptor-associated kinase 4 (IRAK4) to form the myddosome complex, which serves as a platform to phosphorylate IRAK4, IRAK2, and IRAK1. Alterations in the recruitment and oligomerization of TNF receptor-associated factor 6 (TRAF6) and other signaling intermediates then occur, which participate in the activation of NF-*κ*B, MAPK, p38, Janus kinase, extracellular signal-regulated kinase (ERK) and signal transducer and activator of transcription 3 (STAT3) to initiate the transcription of inflammatory cytokines [5, 71, 74]. The synthesis, secretion, activated signal transduction, and subsequent role of IL-1 signaling are shown in Figure 1. The targeted genes of IL-1 include IL-1*α* and IL-1*β* themselves, as well as other inflammatory factors such as IL-6, IL-8, monocyte chemotactic protein 1 (MCP-1)/C-C chemokine ligand 2 (CCL2), and cyclooxygenase-2 (COX-2) [68, 75, 76]. IL-1R1 can also bind to IL-1Ra, which does not produce a signal due to its lack of an IL-1 receptor accessory protein interaction domain, thereby acting as a competitive binding factor to inhibit proinflammatory signaling [66]. IL-1R2 is a membrane-bound or released form of a decoy receptor with an extracellular region similar to that of IL-1R1. But it has a short cytoplasmic domain unable to generate a signal, acting as a molecular trap to block signal generation [5, 10, 77]. IL-1R2 is the key negative regulator of the IL-1 signaling, acting intracellularly, on the cell surface, and extracellularly to inhibit maturation of IL-1*α*/*β*, sequester their active form, or hinder the assembly of signaling complexes [77]. Soluble receptors (sIL-1R1, sIL-1R2, and sIL-1RAcP) present in the circulation can also sequester IL-1 and reduce signal production [78].

## 6. Role of IL-1 Signaling in Breast Cancer Microenvironment

**Figure 2:**
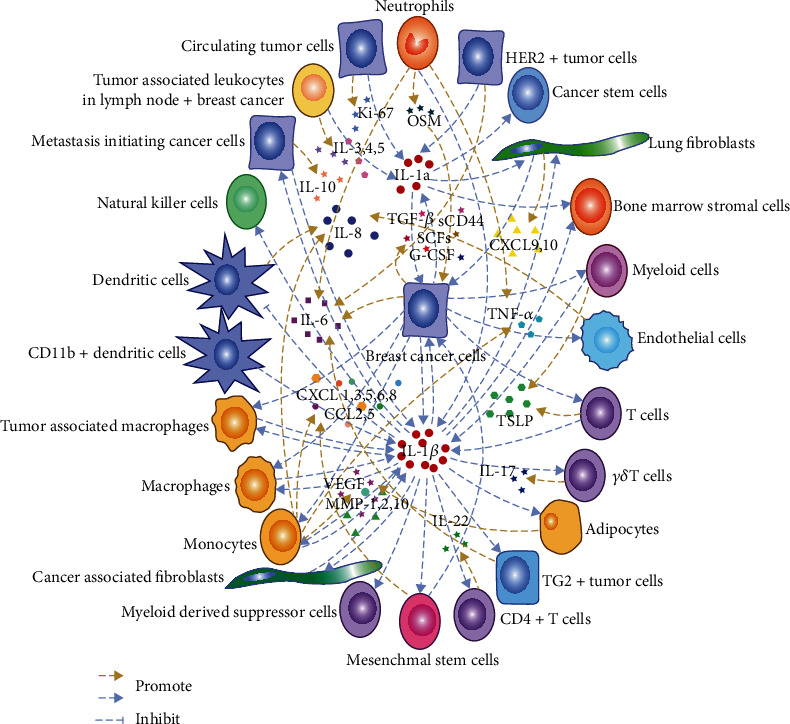
IL-1 signaling-mediated intercellular and intracellular crosstalk in a breast cancer microenvironment. Blue arrows indicate the source and fate of IL-1. Yellow arrows indicate the network of reciprocal influences among different cytokines engaged by IL-1. The source of IL-1 in the breast cancer microenvironment is extensive. Its directions mainly include various immune or inflammatory cells, where it acts to recruit cells and promote differentiation and secretion. Abbreviations: IL—interleukin; MMP—matrix metalloproteinase; TSLP—thymic stromal lymphopoietin; TGF-*β*—transforming growth factor-beta; TNF-*α*—tumor necrosis factor-alpha; sCD4—soluble cluster determinant 4; OSM—oncostatin M; SCFs—stem cell factors; G-CSF—granulocyte-colony stimulating factor; VEGF—vascular endothelial growth factor; CXCL—(C-X-C motif) ligand; CCL—C-C chemokine ligand.

**Figure 3:**
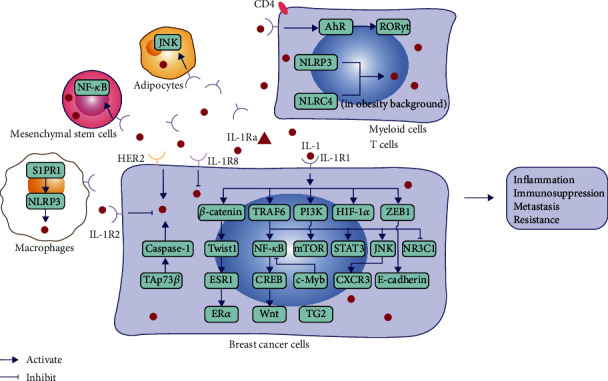
IL-1 signaling-mediated intracellular signal transduction and activation in a breast cancer microenvironment. Abbreviations: IL—interleukin; IL-1R—IL-1 receptor; IL-1Ra—IL-1 receptor antagonist; HER2—human epidermal growth factor receptor 2; ER*α*—estrogen receptor alpha; PI3K—phosphoinositide 3-kinase; mTOR—mammalian/mechanistic target of rapamycin; NF-*κ*B—nuclear factor kappa B; STAT3—signal transducer and activator of transcription 3; AhR—hydrocarbon receptor; ROR*γ*t—retinoid-related orphan nuclear receptor gamma t; CD4—cluster determinant 4; JNK—c-Jun N-terminal kinase; CXCR3—C-X-C chemokine receptor type 3; ZEB1—zinc-finger E-box binding protein 1; HIF-1*α*—hypoxia-inducible transcription factor-1 alpha; Wnt—wingless and int-1; CREB—cyclic AMP response-element binding protein; TG2—transglutaminase 2; TRAF6—TNF receptor-associated factor 6; NLRP3—NOD-like receptor family PYD domain-containing protein 3; NLRC4—NOD-like receptor family CARD-containing protein 4.

As shown in Figures 2 and 3, there is a complex intercellular and intracellular crosstalk mediated by IL-1 signaling in the breast cancer microenvironment, which may contribute to its role in tumor-associated inflammation, immunosuppression during tumor development, metastasis leading to recurrence, and acquired drug resistance.

### 6.1. Formation and Maintenance of Inflammatory Microenvironment

It has been demonstrated that inflammation in cancer is driven by IL-1*β*. “IL-1 signature” is found in patients with HER2- BC [79]. Primary BC cells secrete high levels of the chemokines RANTES/CCL5, CCL2, and granulocyte-colony stimulating factor (G-CSF) that recruit and activate monocytes and instruct them to secrete high levels of IL-1*β* and IL-8. This interaction also promotes the secretion of high levels of matrix metalloproteinase-1 (MMP-1), MMP-2, and MMP-10, ultimately creating a chronic inflammatory microenvironment that supports malignant progression and invasiveness [80]. One of the Th2 inflammatory pathways favoring tumor protection in BC relies on the secretion of IL-1*β* from primary BC induced by T cell cytokines and thymic stromal lymphopoietin (TSLP). Furthermore, IL-1*β* produced by myeloid cells is involved in the activation of inflammasomes by BC cell-derived factors. Breast cancer cell membrane-associated transforming growth factor-beta (TGF-*β*) is required for IL-1*β* production by DCs. IL-1-dependent transcriptional signaling has also been shown in the blood of patients with metastatic BC [81]. BC cell-derived IL-1*α* also induces expression of TSLP from tumor infiltrating myeloid cells, and TSLP, in turn, induces expression of B cell lymphoma-2 (Bcl-2) in tumor cells, promotes tumor cell survival, and skews the TME toward Th2 inflammation, sustaining lung metastatic survival [82]. Inflammasomes are one of the key regulators of IL-1 production. BC cells induce release of IL-1*β* from myeloid and T cells via activation of the NLRP3 inflammasome, and IL-1*β* activates the transcription factors aryl hydrocarbon receptor (AhR) and retinoid-related orphan nuclear receptor gamma t (ROR*γ*t) to induce IL-22 production in memory cluster determinant 4-positive (CD4+) T cells to promote tumor growth [83]. Activation of inflammasomes as well as increased level of IL-1*β* at the primary and metastatic sites promote the infiltration of myeloid cells such as MDSCs and TAMs into the TME [84].

Transactivation p73*β* (TAp73*β*) has been shown to directly activate the positive transcription of caspase-1 and upregulate the expression of pro-IL-1*β* mRNA and IL-1*β* protein, and thus may be important for the regulation of the inflammasomes and inflammation in tumor [85]. In addition, soluble CD44 (sCD44) antigen derived from the TNBC cell membrane triggers the production of macrophage-derived IL-1*β*, regulates the inflammatory TME, and promotes the growth of primary tumor [86]. However, the role of inflammation in HER2-induced tumorigenesis remains controversial. New studies have found that in HER2+ BC, overexpression of HER2 induces the expression and secretion of IL-1*α*, triggers the activation of other signal sequences including IL-6, and stimulates the NF-*κ*B and STAT3 pathways to generate and maintain cancer stem cells (CSCs) and chronic inflammation to promote cancer initiation and progression [87]. In addition, the BC microenvironment in the context of obesity is associated with the increase of tumor infiltrating myeloid cells, which have an activated NLRC4 inflammasome and IL-1*β*, which drive disease progression through activation of c-Jun N-terminal kinase (JNK)-mediated expression of VEGFA and angiogenesis in adipocytes [88]. The level of chronic inflammation usually also means a higher risk of recurrence of BC after primary treatment [87, 89].

### 6.2. Involvement in Tumor Immunosuppression/Escape

The systemic inflammatory cascade is orchestrated through a CCL2–macrophage–IL-1*β*–*γδ*T cell–IL-17–immunosuppressive neutrophil axis in BC. CCL2 recruits C-C chemokine receptor 2-positive (CCR2+) monocytes from the bone marrow to elsewhere in the body and induces their differentiation into macrophages, promoting the expression of IL-1 derived from TAMs. *γδ*T cells are subsequently induced to expand and produce IL-17, promoting the systemic expansion of immunosuppressive neutrophils and formation of metastasis [90]. Whereas IL-1*β* deficiency leads to low levels of CCL2, hinders recruitment of monocytes and, together with low levels of CSF-1, inhibits differentiation of monocytes into macrophages and results in a relatively high proportion of CD11b+ DCs, whose secretion of IL-12 supports antitumor immunity [91]. In addition, upregulation of IL-1R8 in mammary epithelial cell transformation and primary BC decreased IL-1-dependent activation of NF-*κ*B and proinflammatory cytokine production, inhibited activation of NK cells, and promoted M2-like polarization of macrophages, resulting in impaired innate immune sensing and T cell rejection of the TME [92]. Proinflammatory cytokines expressed by primary breast tumors activate an IL-1*β*-dependent innate immune response in innate immune cells infiltrating the microenvironment of distant metastasis-initiating cancer cells (MICs), which may prevent the development of secondary disease and, conversely, primary tumor resection may prompt recurrence [93]. Another study also showed that the expression of IL-1*β* by MICs in BC was significantly associated with longer relapse-free survival and overall survival, while the lack expression of IL-1*β* by MICs are associated with the worst prognosis, and may contribute to tumor immune escape [94]. Thus, IL-*β* secreted by BC cells at the primary and metastatic sites may have a positive effect on tumor immune escape and metastasis suppression, whereas IL-*β* secreted by immune cells infiltrating the TME exerts a detrimental effect. These new evidences suggest a new line of thinking to link the immunosuppressive/escape microenvironment of BC with IL-1 and thus design tumor suppressive approaches.

### 6.3. Promotion of Metastasis

The infiltration of IL-1*β* inflammatory factors can directly promote the metastasis of BC. In TNBC, the increase of IL-1*β* directly affects the invasiveness of tumor cells [95]. IL-1*β* is both transmission supportive and colonization inhibitory. At the metastatic site, IL-1*β* maintains the systemic environment disseminated MICs in an active differentiated state of zinc-finger E-box binding protein 1 (ZEB1), preventing MICs from producing highly proliferative progeny with active E-cadherin [93]. In addition, the cytokine network composed of IL-1 together with other cytokines has a complex role in metastasis of BC cells. IL-6, oncostatin M (OSM), and IL-1*β* are correlative in expression. OSM induces phosphorylation of STAT3, and IL-1*β* promotes phosphorylation of p65 to synergistically induce IL-6 secretion of ER-MDA-MB-231 cells, promoting the onset of acute and chronic inflammation and metastasis [96]. Evaluation of serum samples from BC patients showed significant positive correlations between levels of IL-1*β* and (C-X-C motif) ligand 8 (CXCL8), and between levels of IL-1*β* and sCD200 in controls. Serum levels of sCD200, CXCL8, IL-1*β*, and CRP were significantly higher in early and advanced BC patients compared to controls [97]. Human IL-1*β* induces expression and secretion of stem cell factors (SCFs) in MCF-7 human epithelial BC cells in a manner dependent on the PI3K/mTOR pathway and hypoxia-inducible transcription factor-1alpha (HIF-1*α*) accumulation/activation [98]. IL-1*β* confers stem-cell-like ability of tumor cells to enhance their metastatic potential. However, another study showed that IL-1*β* increased migration of MDA-MB-231 cells, accumulation of HIF-1*α*, upregulation of CXCR1, and expression of CXCL8 and NF-*κ*B under hypoxia. But inhibition of HIF-1*α* had no effect on IL-1*β*-migration of induced hypoxic cells and could not reduce expression of NF-*κ*B and CXCL8. The NF-*κ*B/CXCL8 pathway in a hypoxic microenvironment may play a compensatory role in the IL-1*β*-induced migration of MDA-MB-231 cells [99].

Several studies have shown that a complex interplay between MSCs and BC cells is closely related to the metastatic potential of BC cells. Compared with normal and other subtypes of BC, the highest level of BRCA1-IRIS (hereafter IRIS) expression was observed in TNBC, the cellular necrotic/hypoxic/inflammatory centre of IRIS overexpressing (IRISOE) tumors or the vicinity formed an invasive niche, and IL-1*β* secreted by IRISOE-TNBC cells recruited and activated bone marrow MSCs to secrete CXCL1. CXCL1 enabled IRISOE-TNBC cells to secrete higher levels of CCL2 and VEGF, which recruit and activate TAMs and endothelial cells (ECs), and induce these cells to secrete S100A8/9 and IL-8, respectively. This interaction contributes to the generation of the metastatic precursor of IRISOE-TNBC [100]. Invasive BC cells (MDA-MB-231 cells) activate NF-*κ*B signaling in MSCs by secreting IL-1*β*, inducing and increasing the production of the same chemokines (CXCL1, 3, 5, 6, 8, and CCL2, 5, etc.) as metastatic ER- BC [101]. Cocultured TNBC cells and MSCs/CAFs in the presence or stimulation of TNF-*α* or IL-1*β* showed increased expression of the prometastatic chemokines CXCL8, CCL2, and CCL5, enhanced angiogenesis, migration and invasion of cancer cells, and a significantly enhanced prometastatic phenotype in TME and tumor cells themselves. Among them, CXCL8 plays a key mediating role [102]. Umbilical cord-derived mesenchymal stem cells (UC-MSCs) were cocultured with breast or ovarian cancer cells, and the switched inflammatory UC-MSCs had no obvious effect on the proliferation or apoptosis of the two cancer models, but IL-1*β* produced in an autocrine manner promoted stem-cell-like properties of cancer cells, initiating the formation of a prestem niche [103].

There is a causal relationship between the inflammatory microenvironment and metastasis. Research found that loss of p53, a key regulator of prometastatic neutrophils, induced secretion of Wingless and int-1 (Wnt) ligands from cancer cells, which stimulated TAMs to produce IL-1*β*, which drives systemic inflammation. Pharmacologically and genetically blocking the secretion of Wnt reverses IL-1*β* expression by macrophages and subsequent neutrophilic inflammation, leading to reduced metastasis formation [104]. High expression of the transcription factor c-Myb was found to repress the expression of a set of inflammatory signature genes in BC, including *Ccl2*, *Cxcl1*, *cxcl2*, *cxcl6*, *Cxcl16*, *Icam1*, *Il1a*, *Tnfrsf9*, *Lcn2*, and *Ikbke*, which were denoted as c-Myb-inflammatory signature [105]. It was subsequently found that c-Myb reduced autocrine signal transduction of the NF-*κ*B pathway in BC and the ability of BC cells to migrate and cross the endothelial barrier through inhibition of the expression of IL-1*α*. Overexpression of IL-1*α* as well as the addition of recombinant protein of IL-1*α* activated NF-*κ*B signaling and restored the expression of inflammatory signature genes that were suppressed by c-Myb [53]. Mouse models of BC reflect that periodontal inflammation (PI) and the resulting IL-1*β* promote the expression of CCL5, CXCL12, CCL2, and CXCL5, which in turn recruit MDSCs and macrophages, ultimately creating a premetastatic niche at the site of inflammation [106].

Tumor lymphangiogenesis is associated with metastasis, but the exact mechanism remains unclear. The novel study identified that sphingosine 1-phosphate receptor 1 (S1PR1) signaling in macrophages promoted lymphangiogenesis via NLRP3-dependent IL-1*β* secretion in mouse mammary tumors infiltrated with CD11b^hi^CD206^+^ TAMs. And since IL-1*β* is involved in tumor pathological rather than physiological lymphangiogenesis, the side effects of targeting IL-1*β* to block tumor lymphangiogenesis may be limited [107]. Macrophage-derived caspase-1-dependent IL-1*β* plays an important role in BC cell lymphatic endothelial cell adhesion and migration across endothelial cell barriers [108]. Tumor-associated leukocytes isolated from lymph node+ BC patients secreted 2- to 5-fold more cytokines than lymph node- patients, with the most increased cytokines being thymus and activation-regulated chemokine (TARC/CCL17), IGF-1, IL-3, TNF-*β*, IL-5, G-CSF, IL-4, and IL-1*α*. These cytokines promote epithelial mesenchymal transition (EMT) and BC lymph node metastasis by upregulating TGF-*β* and vimentin, downregulating E-cadherin, and activating epidermal growth factor receptor (EGFR) (Tyr845) and NF-*κ*B/p65 (ser276) signaling [109]. Circulating tumor cells (CTCs) are precursors to the formation of metastatic lesions and, therefore, are also prognostic markers of poor survival in patients with early-stage BC before the initiation of systemic adjuvant therapy and after adjuvant chemotherapy. Studies have found that IL-1*α* is a marker of tumor cells released into the circulation rather than into the lymphatic system [110]. Neutrophils can assist the formation of a precancerous metastatic niche in distant organs of BC due to activated neutrophils escorting CTCs, facilitating the adhesion of CTCs and ECs, and most CTC-associated leukocytes are N2-like neutrophils. Ki-67 expression was higher in disseminated tumor cells derived from CTC-neutrophil clusters compared with independent CTCs. In contrast, CTC-associated neutrophils frequently expressed TNF-*α*, OSM, IL-1*β*, and IL-6, which matched their receptors on the corresponding CTCs [111].

Studies have confirmed the importance of IL-1 signaling in the promotion of BC bone metastasis. Using a clinically relevant humanized mouse model of BC bone metastasis, altered expression of IL-1*β*, IL-1R1, S100A4, cathepsin K (CTSK), secreted phosphoprotein 1 (SPP1), and receptor activator of NF-*κ*B (RANK) in BC cells as they progress from primary tumor to bone metastasis was demonstrated, and these molecules can be used to predict future bone recurrence in BC patients [112]. This model established that the presence and active function of IL-1*β* had an impact on the occurrence of bone metastases. In-depth studies have shown that bone marrow-derived IL-1*β* stimulates bone colonization of BC cells by inducing NF-*κ*B/cyclic AMP response-element binding protein- (CREB-) Wnt signaling and colony formation of CSCs [113]. Furthermore, IL-1*β* produced endogenously by BC cells in primary sites promotes EMT, invasion, migration, and bone colonization. Upon arrival in the bone environment, contact between tumor cells and osteoblasts or myeloid cells increases the secretion of IL-1*β* by all three of these cell types. High concentrations of IL-1 *β* cause increased proliferation of the bone metastatic niche and bone resorption by osteoclasts, stimulating disseminated tumor cells to grow into overt metastases [114]. Additionally, IL-1 is also a differential regulator associated with pain of metastatic cancer in bone [115]. Bone marrow dissemination of BC cells is an early event, but cells can become latently dormant for years before the development of bone metastases [114]. Treatment of bone metastases is not effective, and IL-1 signaling inhibitors may become new adjuvants to inhibit colonization of disseminated cells to metastases.

The lung is also a common metastatic site for BC. IL-1*α* and IL-1*β* secreted by metastatic BC cells induce the production of CXCL9 and CXCL10 by lung fibroblasts through the NF-*κ*B signaling pathway. A small subset of BC cells specifically expressing CXCR3 exhibited tumor-initiating ability when cotransplanted with fibroblasts, driving JNK signaling, increasing expression of IL-1*α*/*β*, forming a supportive metastatic niche, and promoting lung metastatic tumor growth [116]. The inflammasome/IL-1 pathway is an important mechanism in the development of BC lung metastasis, as confirmed by the significant reduction of lung metastasis in inflammasome or caspase-1-deficient mice, and may be related to IL-1*β*-induced expression of CCL2 in macrophages and tumor cells [84]. In vitro invasion assay confirmed that irradiation targeting D2A1 tumor and its microenvironment increased the levels of plasma IL-1*β*, promoted the infiltration of tumor cells and the development of lung metastasis and increased the activity of MMP-2 and MMP9 [117]. Conversely, genetic studies utilizing the mouse mammary tumor virus polyoma middle tumor (MMTV-PyMT) mouse model revealed that IL-1*α*-mediated IL-1R1 signaling inhibits the proliferation, growth, and lung metastasis of BC cells at early stages of tumorigenesis [118]. Therefore, the role of IL-1*α*-mediated IL-1 signaling in BC lung metastasis may be biphasic depending on the stage and context of tumor development.

### 6.4. Involvement in Tumor Resistance

BC is a HR-driven cancer, so many patients are treated with therapies that lower hormone levels or directly block HR, but most will eventually develop therapeutic resistance. A recent study proposed that IL-1 may provide a conserved basal gene expression pattern in HR+ BC cells that mimic HR- BC cells. Sequestome-1 (SQSTM1/p62) is a differentially expressed gene induced by IL-1 in HR+ and HR- BC cells and is required for survival of HR- cells, playing a role in acquired HR-independent survival and therapeutic resistance. P62 binds to and polyubiquitinates TRAF6, leading to transactivation of NF-*κ*B, forming a positive feedback loop inducing production of IL-1*β* and activation of signaling. P62 may also be involved in the crosstalk between IL-1 and glucocorticoid signaling by inhibiting NR3C1, which encodes a glucocorticoid nuclear receptor that suppresses inflammatory gene expression [52]. In addition, IL-1 was found to mediate the inhibition of estrogen receptor *α* (ER*α*) and progesterone receptor (PR) induced by bone marrow stromal cells in ER*α*+/PR+ BC cells, the upregulation of p62/SQSTM1 and autophagy, and the p62-LC3 interaction. Thus, IL-1*β* may depend on the function of p62 and autophagy to confer a viable ER*α*-/PR- molecular phenotype in ER*α*+/PR+ BC cells, and this may underlie endocrine resistance [119]. In HER2+ BC, HER2 induced expression of IL-1*α* and IL-6, which then increased drug-resistance-related CSCs in primary tumor, while blocking IL-1 signaling increased the efficacy of chemotherapy when combined with cisplatin and paclitaxel [87].

In the cell model of BC cells (6D cells) with EMT induced by IL-1*β* through the activation of the IL-1*β*/IL-1R1/*β*-catenin pathway, upregulation of Twist1 resulted in methylation of the ESR1 gene promoter, which significantly reduced the level of ER*α* and increased the resistance to tamoxifen [120]. After IL-1*β*-highly responsive clone (6D cells) from noninvasive MCF-7 BC cells were stimulated by IL-1*β*, the expression of CDKN1A/p21, TP63, small-fiber neuropathy (SFN), and especially BIRC3, was upregulated, which made BC cells resistant to doxorubicin [121]. The IL-1*β*/IL-1R1/*β*-catenin signaling pathway can also upregulate the expression of tumor protein 63 (TP63) isoform ∆Np63*α*, which in turn leads to increased expression of EGFR and phosphatase 1D (Wip1) and decreased DNA damage sensors and ataxia telangiectasia mutated (ATM). This is involved in the enhancement of the cisplatin resistance of BC cells [122]. Furthermore, IL-1*β* induces IL-6 production by transglutaminase 2- (TG2-) expressing MCF-7 cells through NF-*κ*B-, PI3K-, and JNK-dependent mechanisms, ultimately increasing the stem-cell-like phenotype of cancer cells associated with drug resistance [123].

## 7. Targeting IL-1 Signaling for Breast Cancer Treatment

### 7.1. Direct Blockade of IL-1 Signaling

There are currently four known IL-1 blocking biologics: anakinra, canakinumab, gevokizumab, and rilonacept. Anakinra, the recombinant form of the human IL-1Ra, acts by competitively preventing the binding of IL-1*α* and IL-1*β* to IL-1R1 [124]. Canakinumab is a human monoclonal antibody (mAb) specific for IL-1*β* [125]. Gevokizumab is a recombinant humanized allosteric monoclonal antibody that negatively regulates IL-1*β* signaling through an allosteric mechanism [78]. Rilonacept (ril on'a sept), an approved recombinant fusion protein comprising the extracellular portion of human IL-1R1 and IL-1RAcP fused to the Fc portion of human IgG1, binds to and inactivates IL-1, acting as an “IL-1 trap” [126]. Anakinra and canakinumab are currently approved for the treatment of rheumatoid arthritis, familial Mediterranean fever (FMF), cryopyrin-associated periodic syndrome, Still's disease, and gouty arthritis, while gevokizumab does not currently have a specific indication [69, 127, 128]. Among them, canakinumab has been widely used in clinical experiments for lung cancer [129–131]. The protein formulation, i.e., the solution of IL-1Ra (kineret), may have ultra-long-term stability for 10 years and has clinical applications in metastatic BC (NCT01802970) [11, 132]. In addition, there are several other mAbs against two cytokines or their receptors, respectively, such as lutikizumab (ABT-981), a double variable domain Ig that binds to and inhibits both IL-1*α* and IL-1*β* [133]; anti-IL-1*α* Xilonix [134]; Bermekimab, a true human mAb targeting IL-1*α* cloned directly from human B cells (Epstein-Barr virus immortalized) isolated from humans with endogenous anti-IL-1*α* antibodies [135]; two IL-1*β* neutralizing antibodies, RD24 and P2D7KK [136]; and Nidanilimab, an entire humanized mAb against IL-1RAcP. Most of these antibodies are being used in clinical cancer therapy to block IL-1 signaling [11]. These blockers are shown in Figure 1.

Anakinra is an ideal treatment option with a short half-life for patients who have undergone chemotherapy, and it is increasingly used as an adjuvant therapy to reduce inflammation in metastatic cancer [79, 137]. In humanized mouse models of BC bone metastasis, anakinra treatment reduced the number of mice that developed metastases in human bone implants from 57.14% to 0% [112]. Anti-IL-1*β* treatment reduced hindlimb bone metastasis in the spontaneous MDA-MB-231 BH mouse model [113]. Knockout of IL-1R1 (IL-1R1^−/−^), anakinra or canakinumab reduced bone metastasis and the number of tumor cells shed into the circulation [114]. Blocking IL-1R with IL-1Ra inhibited the invasiveness of Hs578t and MDA-MB231 TNBC cells and the development of bone metastasis [95], and it inhibited tumor growth while reducing the accumulation of myeloid cells [84]. Treatment with anti-IL-1*β* antibody attenuates production of IL-6, the stem-like phenotype, and tumor growth and metastasis in TG2+ BC cells [123]. The use of anakinra *in vivo* reduced the production of IL-22 and tumor growth in BC [83]. Anakinra or TGF-*β* neutralizing antibody treatment significantly decreased the production of IL-13, IL-4, IL-17, and TSLP; increased the production of NF or interferon-gamma (IFN*γ*); and suppressed growth of BC [81]. Secretion of IL-1*β* by IRISOE-TNBC cells within the invasive niche initiates a bidirectional effect with MSCs. Anakinra could break these bidirectional interactions; inhibit generation of MSCs, tumor recruitment, and secretion of CXCL1 *in vivo*; and enhance the efficacy of chemotherapy on IRISOE-TNBC, especially on metastasis [100]. Neither prophylactic nor therapeutic administration of anakinra significantly inhibited the growth of MDA-MB-231-IV tumors in bone and reduced the number of mice that developed bone metastases and subcutaneous tumor volume [138]. Anti-IL-1R1 antibody and anakinra treatment inhibits the growth of E0771 tumor in DIO mice [88]. These evidences revealed that anakinra can modulate the BC microenvironment by blocking IL-1 signaling, reducing tumor growth and metastasis. Unfortunately, there have been few studies on rilonacept and gevokizumab and other biologics used to block IL-1 for the treatment of BC except for the human antibody scFv 12H7, one specially prepared specific binder of IL-1RAcP with high affinity, which had growth inhibitory activity against TNBC cells *in vitro* and *in vivo* [139].

Anakinra provides an optimal treatment. The short half-life of subcutaneously injected anakinra is a distinct advantage, allowing oncologists to stop anakinra treatment at the first sign of infection. This is something that cannot be achieved with persistent antibodies, such as canakinumab [79]. But the results of experiments using anakinra alone may limit understanding of the pleiotropic role of IL-1 in BC, as it is not clear how much of its efficacy is due to blocking IL-1*α* and how much is due to blocking IL-1*β* [140]. Because chemotherapy often leads to myelosuppression, and IL-1 blockade therapy can also suppress peripheral blood neutrophils, the risk of infection may be increased when using IL-1 blockade therapy alone or when using anakinra in combination with standard chemotherapy regimens. Therefore, the precise timing and dosage of IL-1 blockade should be determined before application to cancer patients. In this context, modulation of cancer-cell-induced production of IL-1 might be a better option [86].

### 7.2. Blockade of Up- and Downstream Regulatory Signals of IL-1

Blocking IL-1 in tumors has now expanded immensely. Primary mammary tumor growth and lung metastasis were significantly reduced in NLRP3 knockout mice and caspase-1 knockout mice designed to reduce mature IL-1 production [84]. Tumor growth was significantly reduced in caspase-1/11^−/−^ and NLRC4^−/−^ diet-induced obese mice [88]. TGF-*β* neutralizing antibody treatment was able to decrease production of IL-1*β* in humanized mouse tumors [81]. Antibody-mediated neutralization of sCD44 abrogated production of IL-1*β* in macrophages, modulated the tumor inflammatory microenvironment, and inhibited primary tumor growth [86]. IL-1R8 deficiency in the transgenic mouse model of BC (MMTV-neu/IL-1R8^−/−^) delayed tumorigenesis and reduced tumor burden and metastasis [92]. miRNAs are noncoding microRNAs that negatively regulate gene expression, and play important roles in self-renewal, growth, and metastasis of BC cells [141, 142]. miR-146a-5p can downregulate expression of IRAK1 by directly binding to its 3′-untranslated region and inhibit proliferation and invasion of BC cells [143]. NF-*κ*B inhibitor Bay11-7085 reduced basal levels of IL-1*β* and invasiveness of TNBC cells [95]. Furthermore, since IL-1-induced p62 mediated survival and HR treatment resistance of BC cells, the p62 targeting drug verteporfin (visudyne®) was cytotoxic to HR- BC cell lines [52]. These results illustrate that targeting NLRP3, NLRC4, caspase-1, TGF-*β*, sCD44, IL-1R8, IRAK1, and NF-*κ*B, which affect production and activation of IL-1, and IL-1-mediated downstream signaling p62, are also effective ways to modulate IL-1 signaling in the BC microenvironment.

### 7.3. Combination Therapy

It has been mentioned before that anti-IL-1 or anakinra may decrease the resistance of BC and improve the efficacy of chemotherapy. Furthermore, it was shown that the median duration of treatment with anakinra in combination with one of the standard chemotherapeutic agents (albumin-bound paclitaxel, eribulin, or capecitabine) for BC was 4 months in 11 women with metastatic HER2- BC. Gene expression of IL-1*β*, IL-1R1, IL-1R2, and IL-1RAcP, the five members of the TLR family and the IL-1 signaling kinases MyD88 and spleen tyrosine kinase (SYK) were decreased during two weeks of daily anakinra and during the pilot trial. Conversely, the expression of some NK cell and cytotoxic T cell genes that favor immune-mediated tumor destruction was increased [79]. It is suggested that chemotherapy combined with anakinra treatment may also have the effect of restoring antitumor immunity. In addition, anakinra may also enhance the effects of other treatment modalities, such as immunotherapy. Treatment of wild type mice with 4T1 tumors first with anti-IL-1*β* antibody and then with antiprogrammed cell death protein 1 (PD-1) antibody resulted in a therapeutic outcome that differed from the partial growth inhibition resulting from each antibody alone in that, instead, the tumor progression was completely abrogated [91]. Chimeric antigen receptor- (CAR-) T cells are genetically modified T cells with potential to target the TME and treat solid tumors. Toxicities arising in tumor CAR-T therapy, IL-1-mediated inflammation, and IL-1-induced IL-33-mediated anaphylaxis could be suppressed by the anti-inflammatory cytokine IL-37, thereby contributing to the amelioration of adverse effects of CAR-T therapy [144]. Anakinra can reduce the inflammation and immunosuppression caused by IL-1, contributing to the enhanced antitumor activity. More information on these treatments or interventions is shown in Table 1.

The combination with mAbs is a great focus of future development for IL-1-related therapies, both with immune checkpoint inhibitors, and with other molecularly targeted antibody classes of drugs. As tumor-hyperactivated IL-1 signaling is also responsible for the failure of targeted therapies, targeted therapies using monoclonal antibodies in combination with IL-1 blockade might have improved efficacy [145]. There are a number of recent studies that support this inference. For example, inhibition of IL-1R1 reduced the resistance of metastatic colorectal cancer (mCRC) to cetuximab (a monoclonal antibody-targeting EGFR) [146]. IL-1*α* induced a T cell-dependent antitumor immune response increasing the antitumor efficacy of cetuximab against head and neck squamous cell carcinoma (HNSCC) [147]. In a phase II study, the good activity and manageable safety profile of fluorouracil (5-FU) in combination with bevacizumab and anakinra were demonstrated in mCRC patients who did not respond to chemotherapy and antiangiogenic therapy [137]. But such studies are lacking in BC.

As mentioned earlier, fatal systemic inflammation is a drawback of this therapy. IL-1R1 blockade in combination with chemotherapy may also increase toxicity [81]. Therefore, whether anakinra is a safe adjuvant to chemotherapy and other treatments remains to be demonstrated.

## 8. Conclusions and Perspectives

As a key regulatory inflammatory cytokine, IL-1 is produced in response to not only the stimulus of cell damage, necrosis or environmental stress but also the demand of certain tumors, including BC, and in turn it activates downstream and surrounding inflammatory signals that act to recruit, promote inflammation, induce immunosuppression, promote metastasis, and participate in drug resistance, thus providing a favorable environment for tumor survival.

IL-1-mediated inflammatory signaling participates in immunosuppression and immune escape through the production and maintenance of an inflammatory microenvironment, which is conducive to the progress of BC. Therefore, blocking of abnormal IL-1 signaling caused by a tumor can be used as an immunotherapy or adjuvant immunotherapy to reduce inflammation/immunosuppression and enhance antitumor immunity [148]. In the context of BC, the dysregulated expression of genes, transcription factors, inflammatory cytokines, chemokines, and signaling pathway proteins that depend on or involve in the regulation of the production, secretion, and function of IL-1 signaling molecules, as well as the IL-1-mediated crosstalk between tumor cells and tumor infiltrating immune cells plays an important role in determining the prometastatic potential and therapeutic resistance. To date, the role of IL-1 signaling in tumors has been controversial, in part due to differences in cancer contexts, pleiotropic effects of IL-1, and distinct functions of the two IL-1 cytokines. Most of what is currently known about the role of IL-1 signaling in tumors comes from studying the function of individual recombinant or extracellular forms of IL-1 cytokines, actually leading to an inability to determine their relevance to the function of cells as well as established malignant cells, either from cancer patients or from transplantable mouse models [118].

The levels of IL-1 in combination with other cytokines or IL-1*α*/*β* alone in TME or serum in BC patients are correlated with treatment outcome and are likely to be predictive of poorer outcome. The clinical implications of biomarkers that can classify cases in need of action versus those that are best addressed individually are gaining traction [149, 150]. Currently, only a minority of molecules form part of routine molecular diagnosis of BC, and microenvironment-derived biomarkers are potential additions to existing panels of predictive and prognostic markers [35]. Interestingly, a study conducted in 2017-2018 explored the relationship between cognitive function, severity of depressive symptoms, and IL-1 expression in patients with BC treated with systemic anticancer therapy. The protein expression levels of IL-1*α* and IL-1*β* in patients after chemotherapy were significantly lower, and the severity of depressive symptoms was also lower than that before chemotherapy [151]. We need a more detailed understanding of how different types of cells interact in the microenvironment and how IL-1 signaling promotes or suppresses tumors to better use immune cells and IL-1 as targets and biomarkers for BC therapy.

Among the related therapeutic strategies, the results of several *in vivo* and *in vitro* experiments demonstrated the potential of IL-1 as a therapeutic target for metastatic BC. Anakinra is the most widely used FDA approved biological agent for cancer-related inflammation, which targets the BC microenvironment by directly blocking IL-1 signaling, reducing tumor growth and metastasis, and enhancing chemotherapy efficacy. But this therapeutic approach may interfere with the IL-1-mediated innate immunity *in vivo*, which is the biggest limitation. Compared with several other blockers, anakinra mimics the natural mode of IL-1 blockade with more direct apparent effects and representativeness, which may have contributed to the lower cost-effectiveness and use of other blockers in clinical studies. On the other hand, none of the primary aims of various related studies were to investigate the antitumor effects of IL-1 blockade on a targeted basis. We therefore believe that focusing on the antitumor effects of the blockade of IL-1 signaling in future studies is warranted. There are also other candidate targeting strategies in terms of IL-1 signaling blockade, such as IL-1RAcP, IL-1R2/8, inflammasome/caspase-1, IRAK, and NF-*κ*B pathways. At present, BC has a high recurrence rate and rapid disease progression after monotherapy; thus, combination therapy has become a hallmark of BC treatment. Drug combinations using different mechanisms are able to reduce the likelihood of cancer cells developing drug resistance while reducing the therapeutic dose and toxicity of monotherapies [152].

Targeting the tumor microenvironment often requires innovative drug delivery systems, such as nanoformulations, to achieve drug accumulation at the tumor site. How to link nanomedicine to tumor delivery of anti-IL-1 drugs is also a question to be addressed in the future [153]. Altogether, further understanding of the mechanisms by which IL-1 signaling regulates inflammation, immunity, metastasis, and drug resistance in the BC microenvironment, and finding novel targets that are closely related to tumor development and whose blockade does not later have a devastating impact on the role of IL-1 signaling in innate immunity will provide new perspectives for therapeutic strategies in BC, especially in metastatic BC.

## Figures and Tables

**Table 1:** Treatments or interventions targeting or affecting IL-1 signaling in breast cancer.

Treatment/intervention	Targets	*In vivo*/*in vitro*	Models	Findings	References
Anakinra; anti-TGF*β*	IL-1R1	*In vivo*	Hs578T; NOD/SCID/*β*_2_m^−/−^; patients with HER2 metastatic BC (NCT01802970)	Prevented tumor progression and production of IL-13 in humanized mouse model; downregulated specific components of the systemic inflammatory signature observed in patients with metastatic BC and rescued cytotoxic programs thought to be critical for antitumor activity	[81]
Anti-IL-1R; anakinra	IL-1 signaling	*In vivo*	4T1; E0771; BALB/c; C57BL/6	Reduced tumor progression and production of IL-22^+^ cells	[83]
IL1Ra; caspase-1 inhibitor; Ac-YVAD-cmk; anti-IL-1*β*; anticaspase-1; caspase-1^−/−^; NLRP3^−/−^	IL-1 signaling	*In vivo* and *in vitro*	EO771; PyT8; MDA-MB-231; C57BL/6J; NSG; MMTV-PyMT	Reduced tumor growth and lung metastasis accompanied by decreased myeloid cell accumulation	[84]
Anti-CD44	CD44	*In vitro*	MDA-MB231; MDA-MB-468; MCF-7; MCF-10A; 4T1-Luc; THP-1; human serum samples; BALB/c	Abrogated IL1*β* production in macrophages and inhibited growth of primary tumors	[86]
IRAK1 inhibitor synergized with either cisplatin or paclitaxel	IL-1*α* signaling	*In vivo*	FVB/N	Reduction of CSCs and improvement of the chemotherapy efficacy	[87]
Anti-IL-1R1; anakinra; caspase 1/11^−/−^; NLRP3^−/−^; NLRC4^−/−^	NLRC4/IL-1*β*	*In vivo*	Py8119; E0771; C57BL/6N	Reduced tumor growth except NLRP3^−/−^ mice	[88]
Anti-IL-1*β*, anti-PD-1	IL-1*β*, PD-1	*In vivo*	4T1; BALB/c	Anti-IL-1*β* Abs and anti-PD-1 Abs have a synergistic antitumor activity	[91]
IL-1R8^−/−^	IL-1R8	*In vivo* and *in vitro*	HB4a; HB4a-C5.2; NKL; THP-1; MMTV-neu	Reduced tumor growth and metastasis	[92]
IL-1Ra; Bay; Zerumbone	NF-*κ*B signaling pathway	*In vitro*	Hs578T; MDA-MB231	Inhibition of IL-1*β* expression and cell invasiveness	[95]
Anakinra	IL-1R1	*In vivo*	MSCs; IRISOE cell lines; SCID	Decreased recruitment of mouse MSCs into IRISOE-TNBC tumors and their activation to produce and secrete CXCL1	[100]
IL-1Ra	IL-1R1	*In vitro*	MDA-MB-231 and UC-MSCs coculturing system	Blocked prostemness effects of UC-MSCs on cancer cells	[103]
Anakinra	IL-1R1	*In vivo* and *in vitro*	T47D; MCF-7; BB3RC32; BB2RC08; BB6RC37	Reduced bone metastasis	[112]
Sulfasalazine; KG-501	NF-*κ*B; CREB	*In vitro*	MCF-7; MDA-MB-231_BH	Inhibited Wnt-dependent CSC colony formation in the bone environment	[113]
Anti-IL-1*β*	IL-1*β*	*In vivo*	NSG	Reduced tumor formation; increased trabecular bone volume	
IL-1Ra; canakinumab	IL-1*β* signaling	*In vivo*	MDA-MB-231; E0771; NOD/SCID; BALB/c nude	Reduced spontaneous metastasis to human bone	[114]
Caspase-1 inhibitor	Caspase-1	*In vitro*	MDA-MB-231	Abrogated level of transmigration of MDA-MB-231 cells through both blood and lymphatic endothelial cell barriers	
Verteporfin; siRNA-silenced p62	SQSTM1/p62	*In vitro*	MCF-7; MDA-MB-231	Cytotoxic for HR- cell lines	[52]
IRAK1/4 inhibitor; BAY11-7082; SP600125; and LY294002	NF-*κ*B, JNK, PI3K	*In vitro*	MCF-7 (ATCCHTB-22); MCF-7_TG2	Inhibited expression of IL-6 from IL-1*β*-stimulated TG2-overexpressing MCF-7_TG2 BC cells	[123]
Anakinra	IL-1R1	*In vivo*	MDA-MB-231-IV or MCF-7; BALB/c	Reduced growth of tumors in bone and the number of mice that developed bone metastases	[138]
scFv 12H7	IL-1RAcP	*In vivo* and *in vitro*	Patients; MDA-MB-231; HCC-70	Increased expression of IL-1RAcP in both TNBC cell lines and TNBC patient cohort; scFv 12H7 inhibited tumor growth via inhibiting IL-1-activated-NF-*κ*B pathway in TNBC cells	[139]
miR-146a-5p	IRAK1	*In vitro*	MDA-MB-453; MCF-7	Repressive effects on the proliferation and invasion behavior of BC cells by targeting IRAK1	[143]
CAR-T therapy and IL-37	IL-1, IL-33			Inhibited inflammation and toxicity generated in tumor CAR-T therapy	[144]

*Notes*. ^−/−^Symbols indicate that the gene has been knocked out. Abbreviations: CSC—cancer stem cell; MSC—mesenchymal stem cell; NLRP3—NOD-like receptor family pyrin domain domain-containing protein 3; CREB—cyclic AMP response-element binding protein; IRAK1—interleukin-1 receptor-associated kinase 1; TG2—transglutaminase 2; CAR-T—chimeric antigen receptor T cell.
